# A Lanosteryl triterpene from *Protorhus longifolia* augments insulin signaling in type 1 diabetic rats

**DOI:** 10.1186/s12906-018-2337-z

**Published:** 2018-10-01

**Authors:** Mabhida Sihle Ephraim, Johnson Rabia, Ndlovu Musawenkosi, Sangweni Nonhlakanipho Felicia, Louw Johan, Opoku Andrew, Mosa Rebamang Anthony

**Affiliations:** 1 Department of Biochemistry and Microbiology, University of Zululand, Private Bag X1001, KwaDlangezwa, 3886 South Africa; 2 Biomedical Research and Innovation Platform (BRIP), South African Medical Research Council, Tygerberg, 7505 South Africa; 3 Division of Medical Physiology, Faculty of Medicine and Health Sciences, Stellenbosch University, Tygerberg, 7505 South Africa

**Keywords:** Hyperglycemia, Glucose transporter 4, Oxidative stress, Type 1 diabetes and lanosteryl triterpene

## Abstract

**Background:**

A substantial literature supports antidiabetic properties of the lanosteryl triterpene (methyl-3β-hydroxylanosta-9,24-dien-21-oate, RA-3) isolated from *Protorhus longifolia* stem bark. However, the molecular mechanism(s) associated with the antihyperglycemic properties of the triterpene remained to be explored. The current study aimed at investigating the molecular mechanism(s) through which RA-3 improves insulin signaling in streptozotocin-induced type 1 diabetic rats.

**Methods:**

The type 1 diabetic rats were treated daily with a single oral dose of RA-3 (100 mg/kg) for 28 days. The rats were then sacrificed, and blood, skeletal muscle and pancreases were collected for biochemical, protein expression and histological analysis, respectively.

**Results:**

Persistently high blood glucose levels in the diabetic control rats significantly increased expression of IRS-1^Ser307^ while the expression of p-Akt ^Ser473^, p-GSK-3β ^Ser9^, GLUT 4 and GLUT 2 were decreased. However, enhanced muscle insulin sensitivity, which was indicated by a decrease in the expression of IRS-1^ser307^ with a concomitant increase in the p-Akt^Ser473^, p-GSK-3β ^Ser9^, GLUT 4 and GLUT 2 expression were observed in the diabetic rats treated with RA-3. The triterpene-treated animals also showed an improved pancreatic β-cells morphology, along with increased C-peptide levels. An increase in the levels of serum antioxidants such as catalase, superoxide dismutase, and reduced glutathione was noted in the rats treated with the triterpene, while their serum levels of interleukin-6 and malondialdehyde were reduced.

**Conclusions:**

It is apparent that RA-3 is able to improve the insulin signaling in type 1 diabetic rats. Its beta (β)-cells protecting mechanism could be attributed to its ability to alleviate inflammation and oxidative stress in the cells.

## Background

Type 1 diabetes mellitus (T1DM) is a multifactorial disease that is characterized by insulin deficiency due to destruction of the insulin producing pancreatic beta (β)-cells. To date, the exact causes of T1DM remain unknown. However, its onset is linked to an autoimmune attack on the body’s own pancreatic β-cells as a result of environmental stimuli on genetically predisposed individuals. T1DM which is common amongst young children and adults, accounts for 5–10% of diabetes cases world-wide [1, 2]. According to the latest statistical report from the Center of Disease Control and Prevention (CDC, 2017), T1DM affects 80,000 children annually and this number is expected to increase by 1.4% each year [3]. Due to early onset and longer duration of the disease, affected individuals are at increased risk of developing cardiac failure at a young age [4]. This does not only place a financial burden on individual households, but also on the countries’ health system budgets.

Insulin secretion and action are crucial in maintaining glucose homeostasis, which is mediated through translocation of glucose transporters (GLUTs). Skeletal muscle is responsible for 75% of insulin-mediated glucose disposal [5] through activation of the phosphatidylinositol 3-kinase (PI3-K)/protein kinase B (2) (Akt2) pathway and subsequent translocation of GLUT 4 to the plasma membrane [6]. Persistent hyperglycemia or glucotoxicity observed in diabetic patients, as a result of insulin signaling impairment, is the underlying cause of various diabetic complications. This impairment is known to be concomitant to oxidative stress and an augmented pro-inflammatory response [7].

Though regular intravenous insulin injection is used to manage T1DM, this management strategy neither cures nor prevents onset of diabetes-induced complications. Thus, more effective treatment regimens are required in a quest to prevent, delay or more effectively treat the disease. Therefore, there is a growing interest in plant-derived bioactive compounds as potential candidates in the development of drug formulations with multiple targets to combat diabetes and its associated complications.

**Fig. 1:**
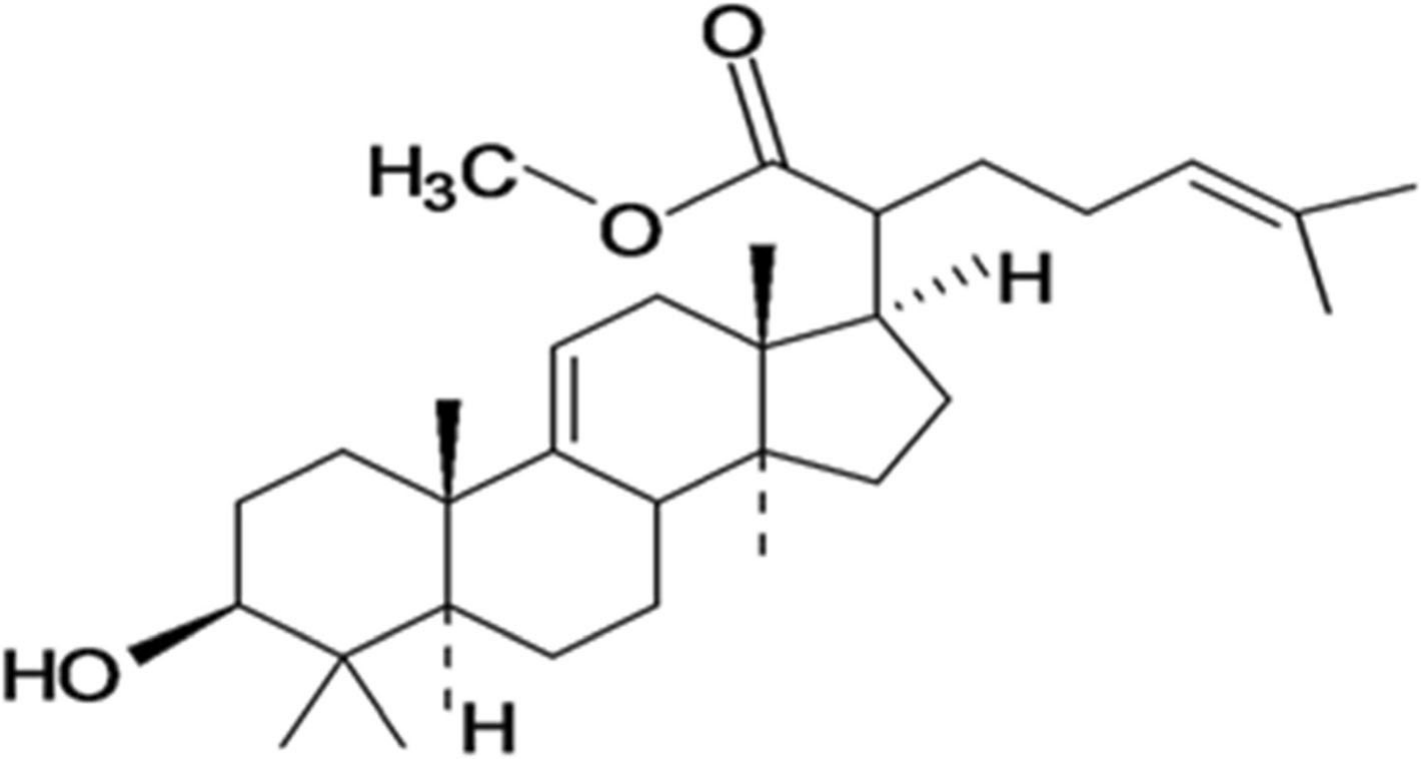
Chemical structure of lanosteryl triterpene (RA-3)

Various in vitro and in vivo studies have shown that triterpenes, a diverse group of natural compounds, have the ability to inhibit intestinal glucose absorption [8–10] and supress raised blood glucose levels through increased insulin secretion and cellular glucose uptake in peripheral tissues [11, 12]. A lanosteryl triterpene (RA-3, Fig. 1) isolated from *Protorhus longifolia* (Benrh.) Engl. (Anacardiaceae) stem bark has been reported to possess hypoglycemic properties, which are also associated with stimulation of cellular glucose uptake [13] and improved glucose tolerance [14, 15]. However, the molecular mechanism(s) associated with these antidiabetic properties of the compound remained to be elucidated. Therefore, this study was set out to explore the molecular mechanism(s) through which RA-3 exerts its hypoglycemic effect. Its effect on peripheral insulin signaling in skeletal muscle of STZ-induced type 1 diabetic rats was investigated in this study.

## Methods

### Extraction and isolation of RA-3

Fresh stem bark of *Protorhus longifolia* (specimen voucher number RA01UZ) was collected from KwaHlabisa, KwaZulu-Natal (KZN), South Africa. The plant was verified by Dr. N.R. Ntuli from the Botany Department, University of Zululand, South Africa. The plant material was then air-dried and ground to powder. The targeted lanosteryl triterpene, RA-3 (Fig. 1), was extracted and isolated from the chloroform extract of *P. longifolia* using chromatographic techniques as previously reported [13, 16]. Spectroscopic (IR, NMR) data analysis was used to confirm the chemical structure of the compound.

### Induction of type 1 diabetes

Approval for use of laboratory animals (*Rattus norvegicus*) and experimental procedures was granted by the University of Zululand research ethics committee (ethical clearance number: UZREC 171110–030 PGM 2016/329). Male *Sprague-Dawley* rats (*n* = 20, 150–200 g) were obtained from the Biochemistry and Microbiology Department, University of Zululand. The animals were maintained under standard conditions (12-h light/dark cycle, relative humidity, ~ 50%, and temperature, 23–25 °C). Before commencement of experimental procedures, rats were acclimatized for five days with free access to water and pelleted rat feed, ad libitum. T1DM was induced by giving the rats a single intraperitoneal injection of streptozotocin (STZ) solution (60 mg/kg). Five days after the STZ injection, blood samples were obtained by tail prick for blood glucose measurement. The fasting blood glucose level was measured with ACCUTREND PLUS® glucometer (Roche Diagnostics, Mannheim, Germany). The rats with the fasting blood glucose levels higher than or equal to 11 mmol/L were considered diabetic and they were used in the study.

### Preparation of RA-3 solution

RA-3 was dissolved in Tween 20 (2%) to prepare a working solution of 100 mg/kg body weight of the rat. The prepared dosage was based on previous studies performed in our laboratory [14, 16].

### Treatment of diabetic animals with RA-3

STZ-induced diabetic rats were randomly divided into three groups with each group consisting of five animals (*n* = 5). The animals were then orally administered with the drugs or carrier solvent as follows: (I) diabetic control group was given Tween 20 (2% diluted with distilled water); (II) diabetic animals treated with RA-3 (100 mg/kg); and (III) diabetic animals treated with metformin (100 mg/kg). The normal control group (IV) was given an equivalent volume of distilled water. The animals received an oral single dose of the RA-3 and metformin (standard antidiabetic drug) daily for 28 days. Fasting blood glucose levels of the rats were measured at seven days intervals using the ACCUTREND PLUS® glucometer (Roche Diagnostics, Mannheim, Germany) for the full duration of the experimental period. Upon completion of the experiment, the animals were fasted overnight, and then placed in an induction chamber were it was anesthetized by inhalation (2% isoflurane mixed with 98% Oxygen).The loss of sensation was further confirmed by the pedal withdrawal reflex before any procedure was performed. Blood from the animals was collected by cardiac puncture for serum analysis. Thereafter, the pancreas, liver and skeletal muscles were harvested for histology and western blot analysis, respectively.

### Biochemical analysis of serum antioxidants, malondialdehyde, interleukin-6 and C-peptide levels

Blood samples were centrifuged (Eppendorf® MiniSpin® 5454 Micro centrifuge, Merck) at 4 °C for 10 min at 1200 rpm. Serum was then collected into fresh tubes and stored at − 80 °C until required. The serum levels of catalase (CAT), superoxide dismutase (SOD), reduced glutathione (GSH) and malondialdehyde (MDA) were measured using commercially available assay kits from Sigma-Aldrich (St. Louis, MO, USA). An enzyme-linked immunosorbent assay (ELISA) kit obtained from Sigma-Aldrich Co. Ltd. (Steinheim, Germany) was used to measure the serum interleukin-6 (IL-6) level. Standard clinical laboratory (Global Clinical & Viral Laboratory, Richards Bay, SA) procedures were followed to measure the serum level of C-peptide.

### Hematoxylin and eosin stain of pancreas

The excised pancreatic tissues were fixed in neutral buffered formalin (10%) until they were required for histological analysis. The formalin fixed tissues were routinely processed by a Leica TP 1020 automated processor (Leica Biosystems, Buffalo Grove, IL, USA) and embedded in paraffin wax. Tissues were sectioned and attached to aminopropyltriethoxysilane coated glass slides, after which they were stained with hematoxylin and eosin [15]. Examination of the slides was performed by an independent Pathologist without prior knowledge of the experimental groups (Vet Diagnostix Laboratories, Pietermaritzburg, South Africa).

### Western blot analysis

To investigate effect of RA-3 on some known proteins involved in insulin signaling pathway, immunoblots against IRS-1^ser307^, p- Akt^Ser473^, p- GSK-3β^Ser9^, GLUT 4 and GLUT 2 were performed. Snap frozen skeletal muscle and liver tissues (100 mg each) were separately lysed in lysis buffer (Pierce Biotechnologies, Rockford, CA, USA) using a tissue lyser. Thereafter, the samples were centrifuged (Eppendorf® MiniSpin® 5454 Micro centrifuge, Merck) at 4 °C for 20 min at 12,000 rpm. Supernatant was collected and stored at − 20 °C until required. Protein (30 μg) was mixed with an equal volume of 2× Laemmli sample buffer (Biorad) before it was denatured at 95 °C. The denatured protein samples (30 μg) were loaded on a 12% SDS-polyacrylamide gel (Bio-Rad, Hercules, CA, USA) and transferred to a polyvinylidene fluoride (PVDF) membrane (Bio-Rad, Hercules, CA, USA) [17] and membranes containing the proteins of interest were then incubated at 4 °C for 16 h with the following primary antibodies: anti- IRS-1^Ser307^ (1:500), phospo-Akt^Ser473^ (1:1000), phospho-GSK-3β^Ser9^ (1:1000) (Cell signaling, Danvers, MA, USA), anti-GLUT 4 (1:1000) (Sigma-Aldrich Chemical Co., St. Louis, MO, USA) and GLUT 2 (1:500) (Sigma-Aldrich Chemical Co., St. Louis, MO, USA). Membranes were then washed and incubated with the appropriate horseradish peroxidase conjugated secondary antibody at room temperature for 90 min. All proteins were normalized to a loading control (β-Actin) (1:500) (Santa Cruz Biotechnology, Dallas, TX, USA). Chemidoc-XRS imager and Quantity One software (Bio-Rad Laboratories, Hercules, CA, USA) were used to detect and quantify the proteins. Image J software was used to quantify the signal intensity of the bands.

### Data analysis

Data were analyzed using GraphPad Prism software (version 5.03). Experiments were performed in triplicates and data were expressed as mean ± SEM. Statistical differences between groups was determine by One-way analysis of variance (ANOVA), followed by Tukey post-hoc test. The values were considered statistically significant where *p* ≤ 0.05.

## Results

### Fasting blood glucose levels of the STZ-induced diabetic rats

Table 1 shows the results of the effect of RA-3 on the fasting blood glucose levels of the STZ-induced diabetic rats following the experimental period of 28 days. Persistently higher fasting plasma glucose levels were observed in the diabetic control rats. However, treatment of the diabetic rats with RA-3 significantly lowered the blood glucose levels by 67%. The observed effect was similar and comparable to the metformin treated group (69%), which served as the positive control group.

**Table 1:** Results of the effect of RA-3 on fasting blood glucose levels of the STZ-induced type 1 diabetic rats

Group	Baseline (mmol/L)	Blood glucose levels Day 28 (mmol/L)	∆Blood glucose levels (%)
Non-diabetic control	4.2 ± 0.22	4.3 ± 0.04	
Diabetic control	14.0 ± 0.58****	27.0 ± 1.14****	
Diabetic + RA-3	13.3 ± 0.58****	4.4 ± 0.44^####^	67
Diabetic + metformin	13.8 ± 0.40****	4.3 ± 0.44^####^	69

Results are expressed as the mean ± SEM, *n* = 5. **** *p* ≤ 0.0001 versus non-diabetic control, ^####^ *p* ≤ 0.0001 versus diabetic control

### Serum antioxidants and C-peptide levels

The effect of RA-3 on serum antioxidants and C-peptide levels in the STZ-induced diabetic animals are presented in Table 2. The STZ-induced type 1 diabetic rats showed a significant decrease in GSH (4.31 ± 0.150, *p* ≤ 0.001), SOD (29 ± 0.040, *p* ≤ 0.01), CAT (0.05 ± 0.006, *p* ≤ 0.05) and C-peptide levels (0.5 ± 0.220, *p* ≤ 0.0001) when compared to the normal control group. Treatment of the animals with either RA-3 or metformin was able to significantly increase the serum glutathione (6.05 ± 0.130, *p* ≤ 0.05), superoxide dismutase (55 ± 0.011, *p* ≤ 0.05) catalase (0.10 ± 0.004, *p* ≤ 0.05) and C-peptide (0.8 ± 1.020, *p* ≤ 0.0001) levels when compared to their diabetic control counterparts.

**Table 2:** Effect of RA-3 on serum antioxidants and C-peptide levels of the diabetic animals

Group	GSH (nmol/mL)	SOD (Inhibition rate %)	CAT (Units/mL)	C-peptide (μg/L)
Non-diabetic control	7.33 ± 0.010	56 ± 0.005	0.12 ± 0.005	0.8 ± 0.010
Diabetic control	4.31 ± 0.150***	29 ± 0.040**	0.05 ± 0.006*	0.5 ± 0.220****
Diabetic + RA-3	6.05 ± 0.130^*#^	55 ± 0.011^#^	0.10 ± 0.004^#^	0.8 ± 1.020^####^
Diabetic + metformin	6.40 ± 0.140^*#^	54 ± 0.012^#^	0.10 ± 0.006^#^	0.8 ± 0.410^####^

Results are expressed as the mean ± SEM, *n* = 5. * *p* ≤ 0.05, ** *p* ≤ 0.01, *** *p* ≤ 0.001, **** *p* ≤ 0.0001 versus non-diabetic control, ^#^ *p* ≤ 0.05, ^####^ *p* ≤ 0.0001 versus diabetic control

### Serum MDA and IL-6

**Fig. 2:**
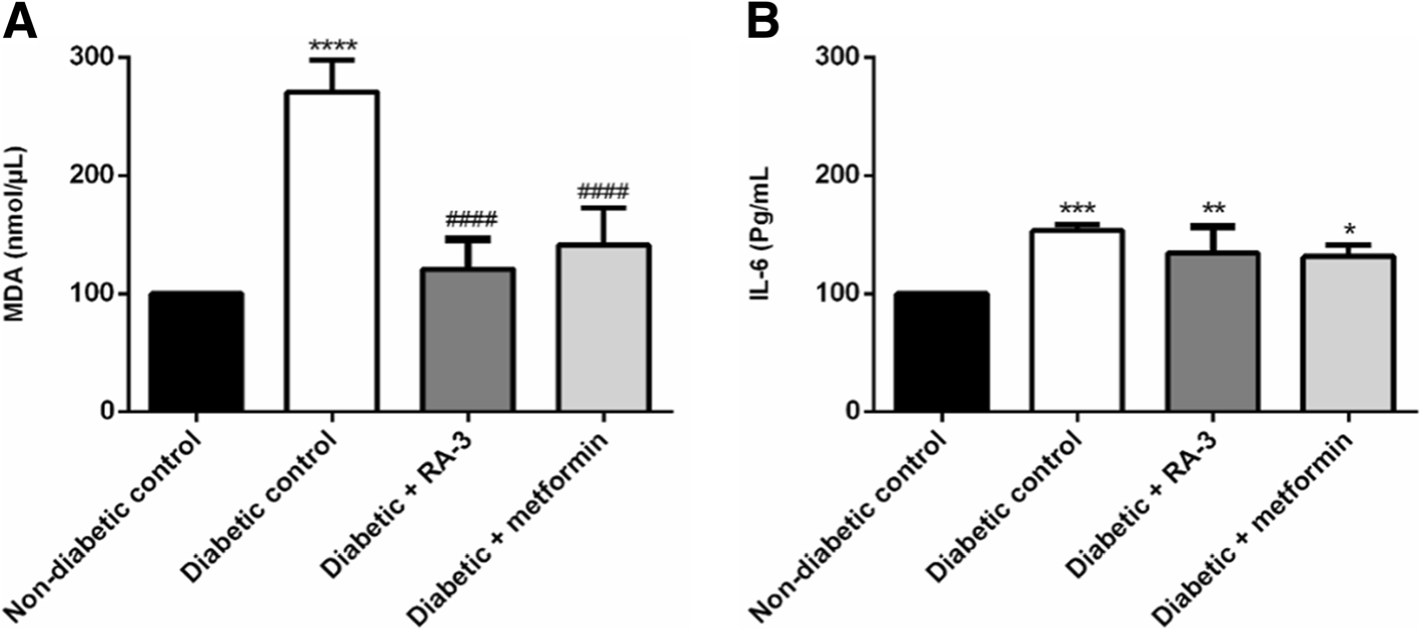
Effect of RA-3 on serum (**a**) MDA and (**b**) IL-6 levels in STZ-induced type 1 diabetic animals. Results are expressed as the mean ± SEM, *n* = 5. * *p* ≤ 0.05, ** *p* ≤ 0.01, *** *p* ≤ 0.001, **** *p* ≤ 0.0001 vs. non-diabetic control, ^####^ *p* ≤ 0.0001 vs. diabetic control

The effect of RA-3 on the serum MDA and IL-6 levels are presented in Fig. 2. The STZ-induced type 1 diabetic rats showed a significant increase of serum MDA (270 ± 13.4%, *p* ≤ 0.0001) and IL-6 (153 ± 2.70%) when compared to the normal control group. After 28 days of treatment, a decreased in the levels of serum MDA (121 ± 12.5%, *p* ≤ 0.0001) and IL-6 (135 ± 10.9%) was observed in the RA-3 treated group as compared to the diabetic control group. This was consistent with the observed increase in antioxidant levels in the RA-3 treated group. A similar and comparable effect was also observed in the metformin treated group. However, the effect of either RA-3 or metformin to reduce IL-6 levels was not significant.

### Effect of RA-3 on the morphology of β-cells of the STZ-induced diabetic rats

**Fig. 3:**
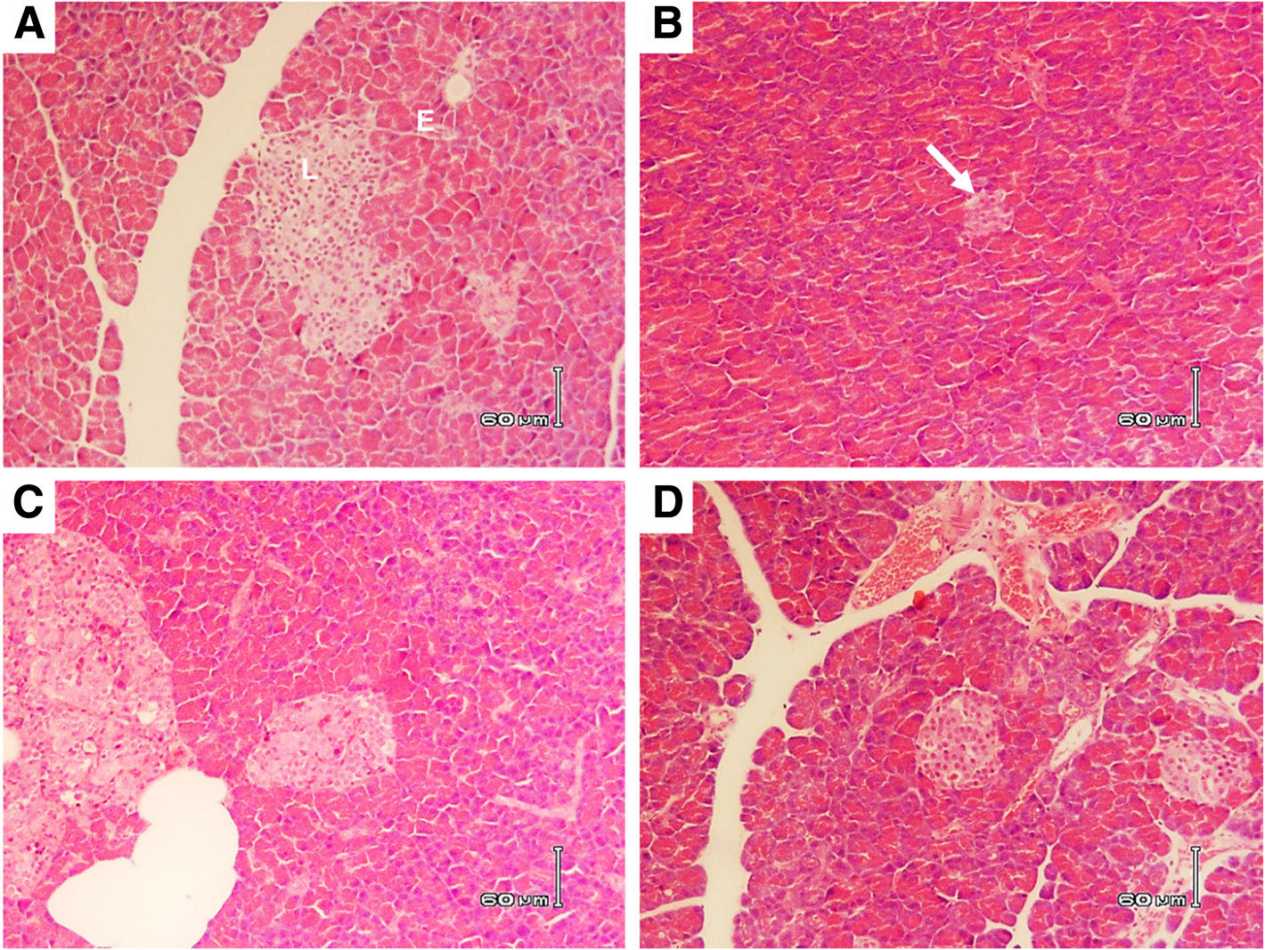
The effect of RA-3 on pancreatic islet morphology of STZ-induced type 1 diabetic rats. **a** Normal control group, (**b**) Untreated diabetic group, (**c**) RA-3 treated group, and (**d**) Metformin treated group. (L) indicates a normal islet without any histological alterations, (E) exocrine portion of pancreatic tissue and an arrow illustrates STZ-induced shrinkage of islets .NB: For each image the magnification (200X). Bar indicator is 60 μm

Histological evaluation of the pancreatic sections from the different experimental groups was performed. The results of the histology are shown in Fig. 3. The pancreas of the normal rats (Fig. 3a) showed normal islet morphology. Whereas the pancreatic tissues from the untreated diabetic group (Fig. 3b) confirmed shrinkage of the islets. However, treatment of the diabetic animals with RA-3 as well as metformin appeared to preserve islet size as shown in Fig. 3c and d, respectively.

### Western blot analysis

**Fig. 4:**
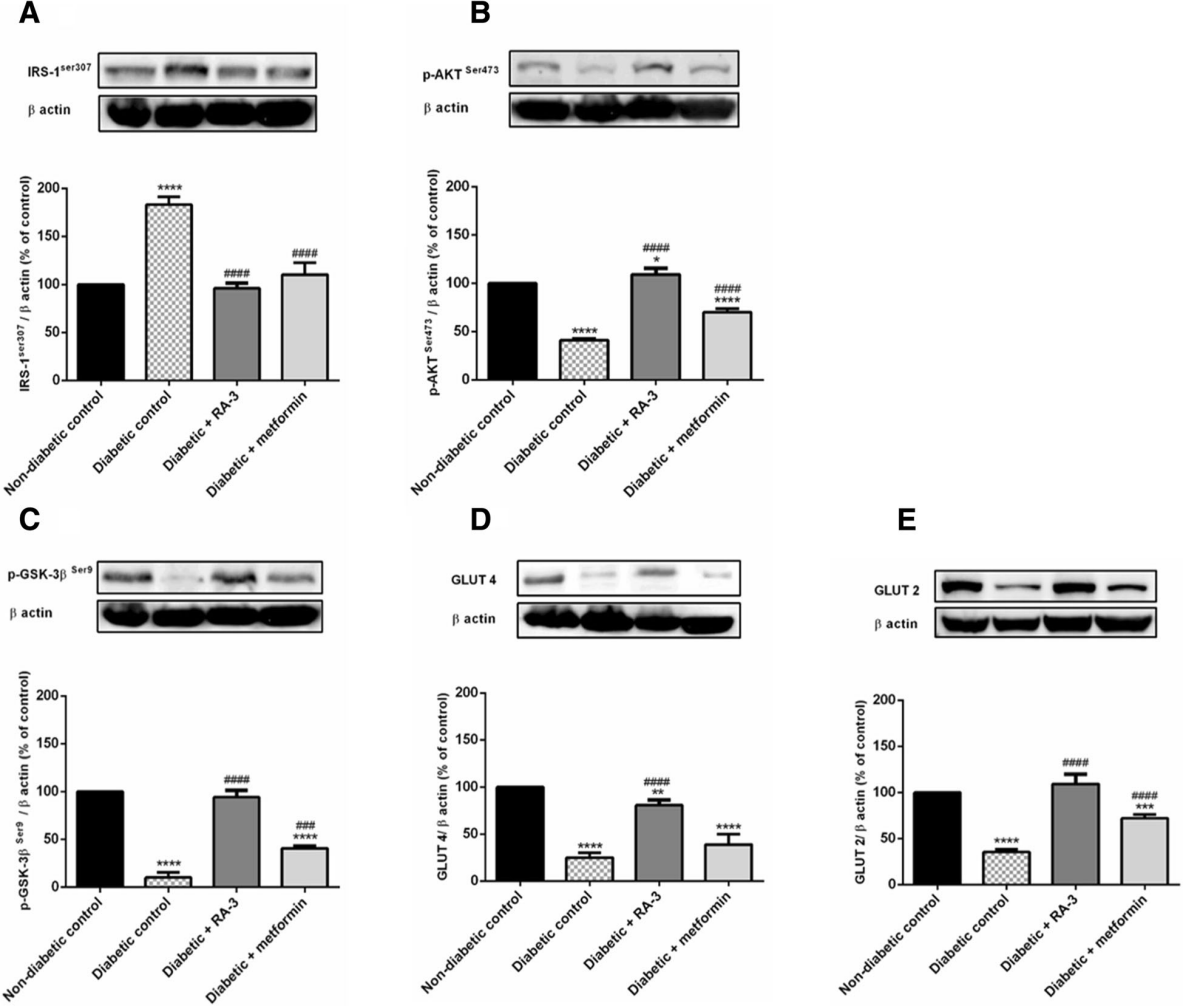
Effect of RA-3 on the expression of IRS-1^ser307^ (**a**), p-Akt^Ser473^ (**b**), p-GSK-3β ^Ser9^ (**c**), GLUT 4 (**d**) and GLUT 2 (**e**) in skeletal muscle tissues of STZ-induced type 1 diabetic rats. Results are expressed as the mean ± SEM, *n* = 5. * *p* ≤ 0.05, **** *p* ≤ 0.0001 vs. non-diabetic control, ^###^ *p* ≤ 0.001, ^####^ *p* ≤ 0.0001 vs. diabetic control

IRS-1^ser307^, p-Akt, p-GSK-3β, GLUT 4 and GLUT 2 are major proteins involved in the insulin-dependent signaling pathway (Fig. 4). The results revealed that high blood glucose levels in the untreated diabetic rats significantly increased the expression of IRS-1^Ser307^ (183 ± 4.06%, *p* ≤ 0.0001), while decreasing expression of p-Akt^Ser473^ (41 ± 0.91%, *p* ≤ 0.0001) and p-GSK-3β ^Ser9^ (10 ± 2.71%, *p* ≤ 0.0001) as compared to the non-diabetic group. The observed effects of high blood glucose levels were reversed following the 28 days treatment of the diabetic rats with RA-3 (96 ± 2.61%, *p* ≤ 0.0001; 109 ± 3.14%, *p* ≤ 0.0001 and 94 ± 3.67%, *p* ≤ 0.0001, respectively) when compared to the diabetic control group. The observed effects were comparable to the metformin treated group. This study showed that while a decrease in GLUT 4 (30 ± 3.97%, *p* ≤ 0.0001) and GLUT 2 (40 ± 1.63%, *p* ≤ 0.0001) expression was observed in the diabetic control group, an increased expression (80 ± 8.16%, *p* ≤ 0.01) and (110 ± 3.14%, *p* ≤ 0.01) of these proteins was observed in the skeletal muscle and the liver tissues of the diabetic animals that received RA-3 (Fig. 4d and e).

## Discussion

Understanding the molecular mechanism that leads to the development and progression of T1DM is an important objective in the management of the disease. Despite the use of current hypoglycemic drugs and insulin injections to manage the disease, the search for new drugs that can prevent and/or effectively treat the disease is on-going. Previous studies have demonstrated the anti-hyperglycemic property of the lanosteryl triterpene (RA-3). This was evidenced by its ability to stimulate cellular glucose uptake in C2C12 myocytes and 3 T3-1 L adipocytes [13] and then improve glucose tolerance in diabetic rats [14, 15]. However, the molecular mechanism(s) through which RA-3 exerts its hypoglycemic effect remained to be explored and understood. Thus, this study investigated potential of RA-3 to augment insulin signaling in the STZ-induced type 1 diabetic rats.

**Fig. 5:**
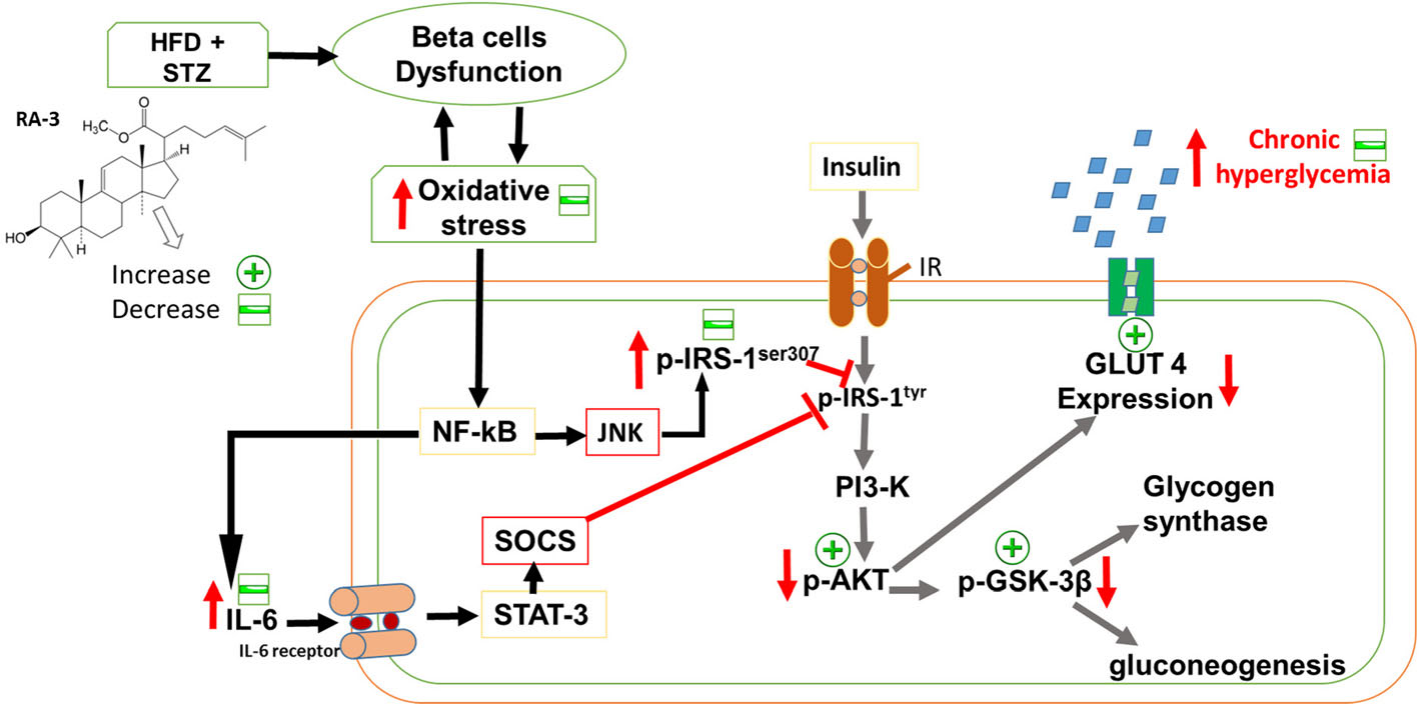
Overview of insulin regulation of major metabolic responses in the cells. Under physiological conditions, insulin binds to its receptor (IR) to enhance positive tyrosine IRS-1 phosphorylation, promoting the activation of PI3-K which in turn phosphorylates and activates p-Akt. High expression of p-Akt activates translocation of glucose transporter 4 and thus increasing glucose uptake by the cell. p-Akt also further phosphorylates GSK-3β, promoting the glucose storage as glycogen. Under pathophysiological conditions, oxidative stress and pro-inflammatory cytokines such as IL-6 enhance negative phosphorylation of IRS-1 ^serine (307)^, causing insulin resistance. Activated NF-_K_B activates serine/threonine kinases (JNK) which phosphorylates IRS-1 and also works via other pathways such as SOCS expression to inhibit the insulin signaling pathway

Insulin stimulates muscle glucose uptake by promoting recruitment of GLUT 4 or GLUT 2 via activation of the phosphatidylinositol 3-kinase (PI3-K) and Akt2 pathway [6]. Under physiological conditions, an increase in the expression of IR-IRS-PI3K-Akt signaling cascade is observed. However, the expression of these proteins is down regulated in the diabetic state. The results from the current study showed that the hypoglycemic effect of RA-3 could partly be linked to its ability to augment insulin signalling (Fig. 5). This is indicated by the observed decrease in IRS-1^Ser307^ expression and increase in p-Akt and p-GSK-3β expression, which was well correlated with the increased expression of GLUT 4 and GLUT 2 in the RA-3 treated group. Increased expression of GLUT 4 and GLUT 2 expression was further supported by the lower blood glucose levels in the RA-3 treated diabetic animals. A pentacyclic triterpene, oleanolic acid, has also been reported to enhance the insulin signaling pathway in the skeletal muscle of STZ-induced diabetic rats [18]. High expression of p-Akt activates translocation of GLUT 4 and thus increases glucose uptake by the cell. p-Akt also further phosphorylates GSK-3β, promoting the glucose storage as glycogen. The ability of RA-3 to increase the expression of p-GSK-3β shows potential of this compound to control glucose homeostasis. These results further support the previous report in which RA-3 inhibited glucose-6-phosphatase and increased hepatic glycogen storage in the STZ-induced diabetic rats [14].

Effective antidiabetic therapeutics should also possess pancreatic β-cells protective properties in order to maintain their production of insulin. RA-3 also showed potential to improve morphology of the pancreatic β-cells of the diabetic rats. The potential β-cells protective effect of the triterpene could be attributed to its ability to increase tissue antioxidant status which was evidenced by increases in GSH, CAT and SOD serum levels in the RA-3 treated group. The decrease in serum MDA levels, a marker of lipid peroxidation, and IL-6 further supported the antioxidant protective potential of the compound. The observed increase in serum C-peptide levels, a reliable indicator of well-functioning pancreatic β-cells [19], further supported the potential β-cells protective effect of the triterpene. The ability of the lanosteryl triterpene to improve pancreatic β-cells morphology and thus increase serum C-peptide levels has recently been demonstrated in type 2 diabetic rats [15]. Furthermore, since oxidative stress and pro-inflammatory cytokines such as IL-6 are implicated in the insulin signaling impairment and β-cells dysfunction [20], the antioxidant and anti-inflammatory effects mediated by RA-3 further support its β-cells protective effect and stimulation of cellular glucose uptake.

## Conclusions

The present study provides evidence that the anti-hyperglycaemic activity of RA-3 could be linked to its ability to protect pancreatic β-cells and improve insulin signaling in skeletal muscles of the diabetic rats. This is indicated by increased expression of proteins involved in insulin signaling and eventual increased expression of GLUT 4 in the muscle of the diabetic animals treated with RA-3. The lanosteryl triterpene also reduced oxidative stress and inflammation which are important mediators of cellular insulin resistance, pancreatic β-cells dysfunction and tissue damage in the diabetic animals. Thus, future research directions, which are also important in addressing limitations of the present study, involve immunohistochemistry of the skeletal muscle to confirm the effect of the triterpene on the insulin signalling in skeletal muscle, unravelling molecular mechanisms through which RA-3 improves the pancreatic islets size and quantification of the number of islet cells in the diabetic rats. Furthermore, the consistent similar results exhibited by RA-3 and metformin, a standard antidiabetic drug, suggests a need to evaluate the antidiabetic effects of a combination of the two drugs.

## Abbreviations

Akt2: protein kinase B (2)
GLUT 4: Glucose transporter 4
GLUTs: Glucose transporters
GSK-3β: Glycogen synthase kinase 3 beta
IL-6: Interleukin-6
IR: Insulin receptor
IRS-1: Insulin receptor substrate 1
PI3-K: Phosphatidylinositol 3-kinase
RA-3: Methyl-3β-hydroxylanosta-9,24-dien-21-oate
STZ: Streptozotocin
T1DM: Type 1 diabetes mellitus
