# Correction to: A Lanosteryl triterpene from *Protorhus longifolia* augments insulin signaling in type 1 diabetic rats

**DOI:** 10.1186/s12906-018-2376-5

**Published:** 2018-12-04

**Authors:** Sihle Ephraim Mabhida, Rabia Johnson, Musawenkosi Ndlovu, Nonhlakanipho Felicia Sangweni, Johan Louw, Andrew Opoku, Rebamang Anthony Mosa

**Affiliations:** 1 Department of Biochemistry and Microbiology, University of Zululand, Private Bag X1001, KwaDlangezwa, 3886 South Africa; 2 Biomedical Research and Innovation Platform (BRIP), South African Medical Research Council, Tygerberg, 7505 South Africa; 3 Division of Medical Physiology, Faculty of Medicine and Health Sciences, Stellenbosch University, Tygerberg, 7505 South Africa

## Correction to: BMC complementary and alternative medicine (2018) 18:265 DOI: 10.1186/s12906-018-2337-z

Following publication of the original article [1], the author reported that the first names and last names of all authors were reversed. The original article has been corrected.

**Incorrect names in the original article:**

1. Mabhida Sihle Ephraim
2. Johnson Rabia
3. Ndlovu Musawenkosi
4. Sangweni Nonhlakanipho Felicia
5. Louw Johan
6. Opoku Andrew
7. Mosa Rebamang Anthony

**Correct names:**

1. Sihle Ephraim Mabhida
2. Rabia Johnson
3. Musawenkosi Ndlovu
4. Nonhlakanipho Felicia Sangweni
5. Johan Louw
6. Andrew Opoku
7. Rebamang Anthony Mosa
